# Single-cell RNA sequencing reveals the role of immune-related autophagy in spinal cord injury in rats

**DOI:** 10.3389/fimmu.2022.987344

**Published:** 2022-09-21

**Authors:** Erliang Li, Rongbao Yan, Kang Yan, Rui Zhang, Qian Zhang, Peng Zou, Huimei Wang, Huan Qiao, Shuang Li, Qiong Ma, Bo Liao

**Affiliations:** ^1^ Department of Orthopaedics, The Second Affiliated Hospital of Air Force Military Medical University, Xi’an, China; ^2^ Department of Orthopaedics, The First Affiliated Hospital of Harbin Medical University, Harbin, China; ^3^ Department of Pathology, Zhongshan Hospital, Fudan University, Shanghai, China

**Keywords:** single-cell RNA sequencing, immune, autophagy, spinal cord injury, microglia

## Abstract

Spinal cord injury refers to damage to the spinal cord due to trauma, disease, or degeneration; and the number of new cases is increasing yearly. Significant cellular changes are known to occur in the area of spinal cord injury. However, changes in cellular composition, trajectory of cell development, and intercellular communication in the injured area remain unclear. Here, we used single-cell RNA sequencing to evaluate almost all the cell types that constitute the site of spinal cord injury in rats. In addition to mapping the cells of the injured area, we screened the expression of immune autophagy-related factors in cells and identified signaling pathways by the measuring the expression of the receptor−ligand pairs to regulate specific cell interactions during autophagy after spinal cord injury. Our data set is a valuable resource that provides new insights into the pathobiology of spinal cord injury and other traumatic diseases of the central nervous system.

## Introduction

Spinal cord injury (SCI) refers to damage to the spinal cord due to trauma, disease, or degeneration with no cure at present (1). Based on data from the National Spinal Cord Injury Statistical Center (NSCISC, USA), the number of new SCI cases increased from 12,000 in 2012 (2) to 17,810 in 2021 (3). However, available treatments for SCI remain limited and unsatisfactory (4). The main reason for this lies in the unique pathophysiological mechanism of SCI (5), and SCI activates multiple processes that occur in the manner in which the degree of injury is defined. A previous study (6) indicated that a range of pathological processes and destruction of the spinal cord structure, occur after SCI, which can lead to edema, inflammation, cell death, demyelination, and remyelination. The pathology of different disease models for SCI is driven by different cell types throughout the spinal cord (7), including microglia, macrophages, and immune cells. Microglia help to maintain local homeostasis (8), and are immediately activated together with macrophages to generate innate immune responses (9). The role and significance of many pathophysiological mechanisms related to SCI have been widely studied from different studied, including autophagy (10, 11), apoptosis (10, 12), pyroptosis (13) and ferroptosis (14). However, the role played by changes in local cellular composition, trajectory of cell development, and intercellular communication at the single-cell level remain unclear.

Emerging technologies have been used to carry out comprehensive personalized analysis of samples with SCI at the genome (15), immunome (16), proteome (17), metabolome (18), and microbiome levels (19). However, differences between single cells can also have profound functional effects. Single-cell RNA sequencing (scRNA-seq) reveals further biological functions by analyzing the transcriptome range of single-cells (20, 21), and allows for unbiased analysis of cell population profiles within injured tissues. Previous studies have analyzed cell-level differences at different times after SCI occurrence in mice (22), and the role of microglia in immune system processes in (23). However, the mechanism underlying immune-associated autophagy in SCI remains unclear. Rats are not simply ‘big mice’; although they are similar in many ways, there are fundamental differences, especially in neuroscience and behavioral research (24). Similarly, rats are preferable to mice for modeling human SCI (25). To our knowledge, this is the first study to performed scRNA-seq analysis of SCI in rats.

The data set created in this study comprises scRNA-seq analysis of all cell types involved in SCI. We constructed the cell map and described the cell heterogeneity of different degrees of SCI; identified the subsets of immune cells, macrophages/microglia, and T cells in different SCI states; and explored the possible relationship between different cells and the role of autophagy in SCI. This high-throughput, multiangle study of SCI could provide novel, comprehensive, and exciting insights into SCI for the development of precise treatment.

## Materials and methods

### Animals

For sequencing and histological validation of tip cells, female Wistar rats [SPF Biotechnology Co., Ltd., Beijing, China; certificate no. SCXK (Jing) 2019-0010] were reared in the Orthopedic Laboratory of the Second Affiliated Hospital of Air Force Military Medical University [certificate no. SCXK (Shaanxi) 2020-007]. The feeding conditions were as previously described (26). All experimental procedures were approved by the Animal and Ethics Committee of the Experimental Animal Center of Air Force Medical University (No. IACUC-20201003).

### SCI surgical procedures

All animals were randomly divided into the following groups. The randomization method has been previously described (26) and is detailed in the Supplementary Information.

### ScRNA-seq combined with bulk RNA sequencing

Spinal cord tissue was collected, and an RNA-seq data set (GSE115067) (27) was obtained for quality control, normalization and data integration, cell clustering, and cell type identification. We performed the following analyses: single-cell subgroup; quasi-sequential; enrichment; gene set variation; intercellular communication; cell score and Regulon regulation based on immune-related autophagy factors (IRAFs); and estimation of the fraction of immune cell types. The methods are detailed in the Supplementary Information.

### Quantitative polymerase chain reaction

Total RNA from the spinal cord was extracted using the M5 HiPer Universal RNA Mini Kit (Mei5bio, Beijing, China) following the manufacturer’s instructions, as detailed in the Supplementary Information.

### Transmission electron microscopy

Autophagy activation was determined by transmission electron microscopy (TEM) analysis of autophagy-related vesicles as detailed in the Supplementary Information.

### Immunostaining

Immunofluorescence staining has been described in detailed in previous studies (26). Details regarding the antibodies, staining conditions and scoring methods are provided in the Supplementary Information.

### Statistical analysis

Data are presented as mean ± standard error of the mean. Student’s t-test was used to compare two groups. One-way ANOVA was used to compare more than two groups. Correlation analysis was used to determine the relationships between independent variables. Statistical significance was set at P < 0.05.

## Results

### ScRNA-seq revealed a high degree of cellular heterogeneity in SCI cells

**Figure 1:**
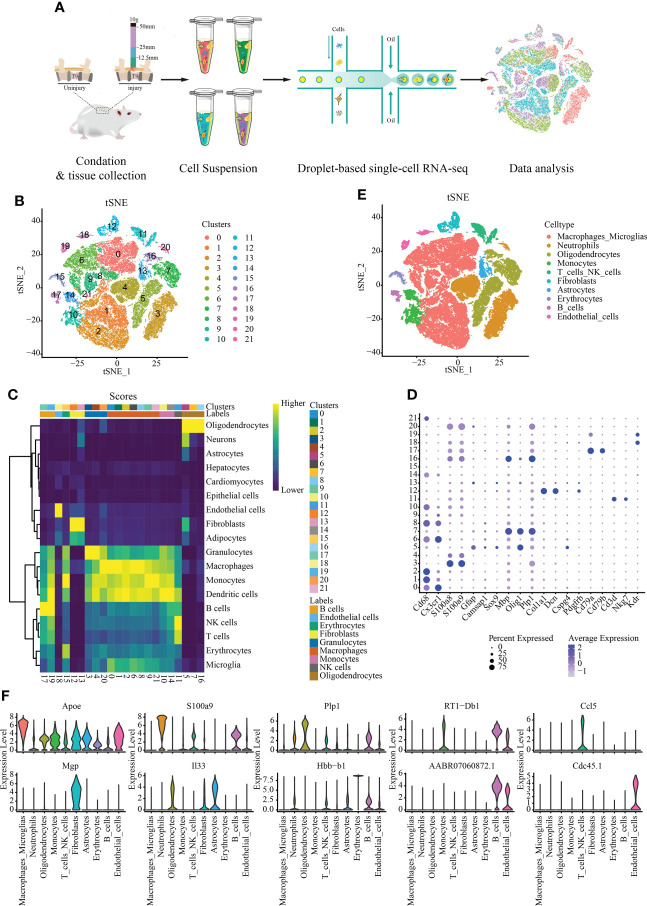
Single-cell data set with reduced dimension clustering and cell type identification. **(A)** Graphical representation of the experimental workflow. **(B)** Cluster analysis of cell groups was carried out, and t-distributed stochastic neighbor embedding (t-SNE) distribution showing cluster analysis groupings. **(C)** Heat map showing cell clusters annotated using SingleR. **(D)** Bubble chart shows the expression of marker genes in each cell cluster. The circle size represents the proportion of gene expression in the cell cluster. Color intensity represents average gene expression. **(E)** t-SNE distribution of different cell types. **(F)** Relative marker gene expression among different cell types. The most highly expressed genes in each cell type is displayed. The abscissa represents the cell type, and the ordinate indicates the expression level.

We sequenced eight spinal cord with different degrees of SCI in rats were sequenced by single cell RNA. Approximately 56,287 cells were obtained after filtration according to the quality control standards (**Figure 1A**) (**Supplementary Figure S1**). After logarithmic standardization, the top 4,000 hypervariable genes were extracted for principle component analysis (PCA) dimensionality reduction. After normalizing of the data, we used the first 15 principal components to cluster the cells with similar gene expression profiles. t-SNE dimensionality reduction was used to visualize 22 independent clusters (**Figure 1B**). Using rat and mouse homologous genes, we identified the cell type of each cluster *via* the mouse transcriptome sequencing data set in SingleR (**Figure 1C**), and the bubble chart shows the expression of marker genes in each cell cluster (**Figure 1D**). Finally, ten cell types were identified: macrophages/microglia, neutrophils, oligodendrocytes, monocytes, T cells/NK cells, fibroblasts, astrocytes, erythrocytes, B cells, and endothelial cells (**Figure 1E**) (**Supplementary Table S1**). A total of 29,197 cells in clusters 0, 1, 2, 6, 8, 9, and 21 were annotated as macrophages/microglia, accounting for 51.872% of all cells analyzed. Additionally, we annotated the clusters: 3, 4, and 20 are annotated as neutrophils (10037, 17.832%); 5, 7, and 16 as oligodendrocytes (7636, 13.566%); 10 and 14 as monocytes (2731, 4.852%); 11 as T/NK cells (1733, 3.079%); 12 as fibroblasts (1388, 2.466%); 13 astrocytes (1191, 2.116%). 18 and Cluster 19 as endothelial cells (1010, 1.794%); 15 as red blood cells (809, 1.437%) and 17 as B cells (555, 0.986%). The most highly expressed differential gene of each cell type was visualized using a violin map (**Figure 1F**).

### Characterization of IRAFs specificity

**Figure 2:**
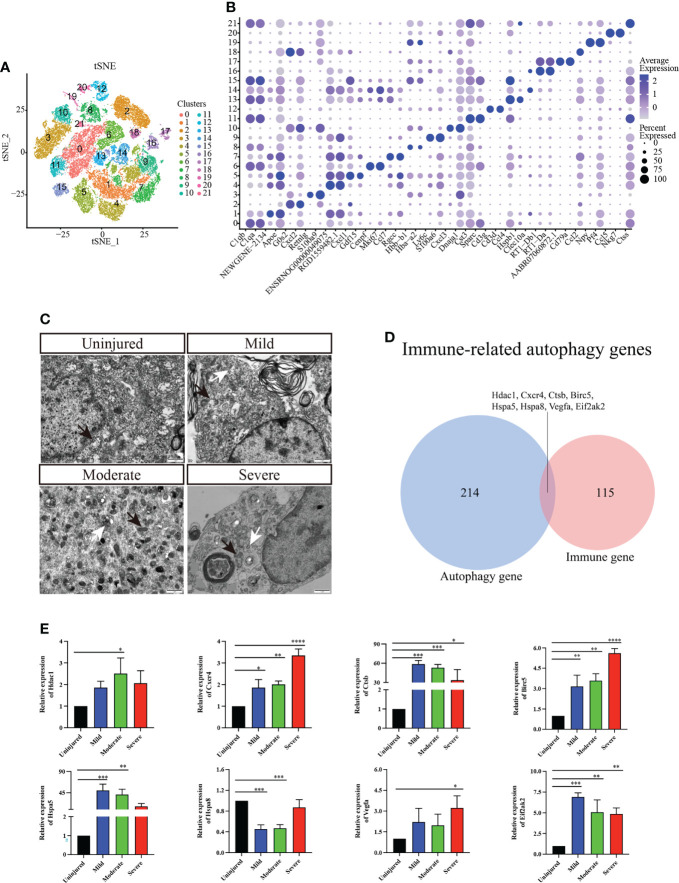
Immunocyte subgroup analysis and pseudosequential analysis. **(A)** t-distributed stochastic neighbor embedding (t-SNE) distribution of different cell clusters. **(B)** Bubble diagram showing the differentially expressed genes among different clusters and display the two most highly expressed genes of each cluster by bubble diagram. **(C)** Electron transmission microscopy of spinal cord tissue. Magnification, 5000X. Scale bars, 1 μm. White arrows indicate autophagosomes and black arrows indicate mitochondria. **(D)** Venn diagram of immune-related autophagic factors. **(E)** Effect of changes in mRNA expression of IRAFs Hdac1, Cxcr4, Ctsb, Birc5, Hspa5, Hspa8, Vegfa, and Eif2ak2 (real-time qPCR) in the injured rat spinal cords. The mRNA expression levels were calculated using the 2^-ΔΔCt^ analysis method. *P < 0.05, **P < 0.01, ***P < 0.001, vs. the control group. Data are expressed as the mean ± SD (n = 3; one-way analysis of variance and Tukey’s post-hoc test). The experiment was repeated in triplicate. ****P<0.0001.

We conducted further cluster analyses of macrophages/microglia, monocytes, neutrophils, T_NK cells and B cells to further understand the heterogeneity of immune cell populations. A total of 22 independent clusters were obtained using the same analysis method (**Figure 2A**), and the differences between cell clusters were analyzed (**Figure 2B**). Next, we evaluated autophagy in different groups of spinal cord tissues using TEM and confirmed that autophagy was activated in the spinal cord tissue (**Figure 2C**).

A total of 123 immune-related differential genes were screened by differential analysis of immune cell subsets, and the top16 were visualized using a violin graph (**Supplementary Figure S2**). Eight IRAFs (Hdac1, Cxcr4, Ctsb, Birc5, Hspa5, Hspa8, Vegfa and Eif2ak2) were obtained of autophagy-related homologous genes (**Figure 2D**). The pseudo-sequence diagram is colored based on two aspects: the pseudo-time process and the stage of the cell population (**Supplementary Figure S3**).

To further clarify the expression of the eight IRAFs in different tissues, we conducted qPCR analyses. We found that different degrees of SCI differentially activated certain IRAFs (P < 0.05). Notably, Hdac1 was significantly upregulated in the moderate group compared to the uninjured group (P < 0.05), and Vegfa was significantly upregulated in the severe group compared to the uninjured group (P < 0.05). However, in both the mild and moderate groups, the expression of Hspa8 was lower than that in the uninjured group (P < 0.001) (**Figure 2E**). We used SCENIC software to identify the co-expression module (regulon) between the transcription factors and the potential target gene (regulon) and the regulon activity score of each cell (regulon activity score, RAS). Regulons related to Tfeb, Usf2 and Spil had relatively high RAS activity in cluster 0, 1, 2, and 6, which were recognized as macrophages/microglia (**Supplementary Figure S4**). This result suggests that IRAFs are involved in the pathophysiological responses induced by different degrees of SCI.

### Macrophage/microglia subsets show tissue-specific patterns

**Figure 3:**
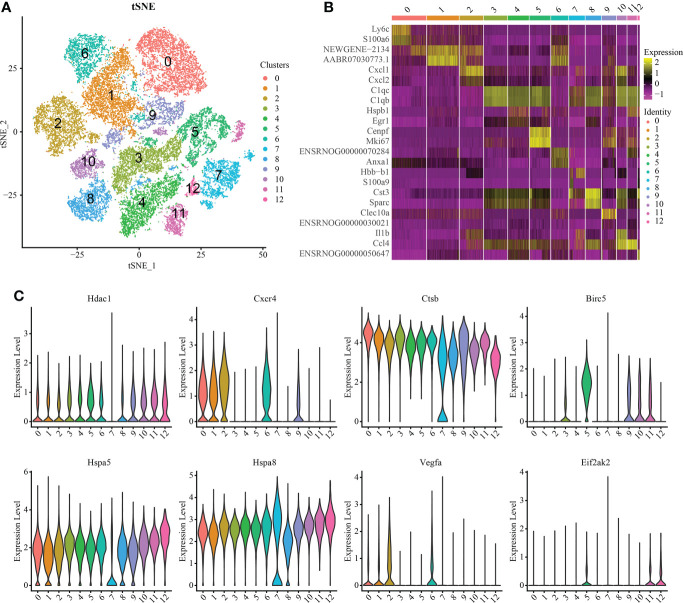
Macrophage/microglia subset analysis and pseudosequential analysis. **(A)** t-distributed stochastic neighbor embedding (t-SNE) distribution of different cell clusters. **(B)** Heat map showing the differential genes among each cluster, displaying the two most highly expressed genes of each cluster. **(C)** Violin map showing the expression pattern of IRAFs in macrophage/microglia subsets.

Macrophages/microglia are particularly important in the process of inflammation. Therefore, we carried out further subgroup analysis of this cell group and obtained a total of 13 independent clusters using the same analysis method (**Figure 3A**). Differences between cell clusters were then analyzed (**Figure 3B**). By labeling microglia with Cd68, we found that the expression of Lc3b in microglia increased with injury severity (P < 0.05) (**Supplementary Figure S5**). To determine the expression of IRAFs in microglial subsets, we also observed the expression of IRAFs among subpopulations in spinal cord tissue subpopulations with different degrees of SCI using violin map. We found that Ctsb,Hspa5 and Hspa8 were expressed in almost all subgroups, while Eif2ak2 was expressed the least (**Figure 3C**).

### T-cell subsets show tissue-specific patterns

**Figure 4:**
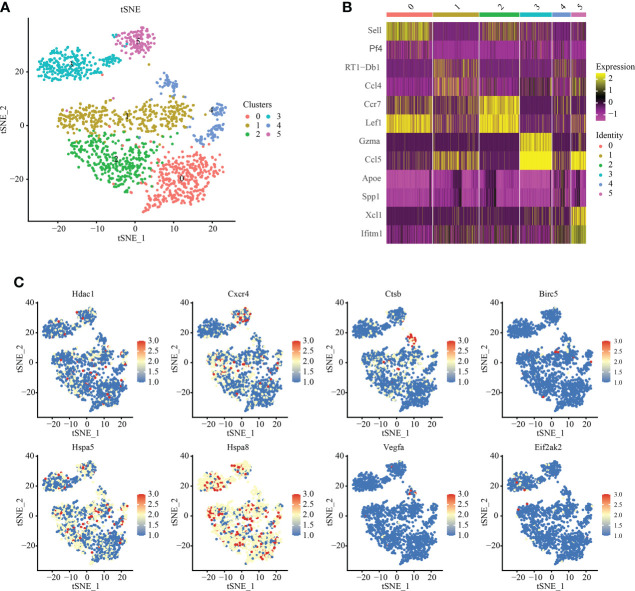
T-cell subset analysis and pseudosequential analysis. **(A)** t-distributed stochastic neighbor embedding (t-SNE) distribution of different cell clusters. **(B)** Heat map showing the differential genes among each cluster, displaying the two most highly expressed genes of each cluster. **(C)** Feature plot showing the expression pattern of IRAFs in T cell subsets.

We analyzed T cell subsets and obtained six independent clusters to better understand the heterogeneity of T cells (**Figure 4A**). Among them, Among them, an expression gene was shared by clusters 0 and 2, and another by clusters 1 and 3, indicating two independent subgroups (**Figure 4B**). We used thermography to measure the expression of IRAFs in all T cells of spinal cord tissue with different degrees of SCI. We found that the expression of Hspa8 was the highest among the IRAFs and was expressed in almost all subsets (**Figure 4C**).

### Intercellular communication in a single-cell group

**Figure 5:**
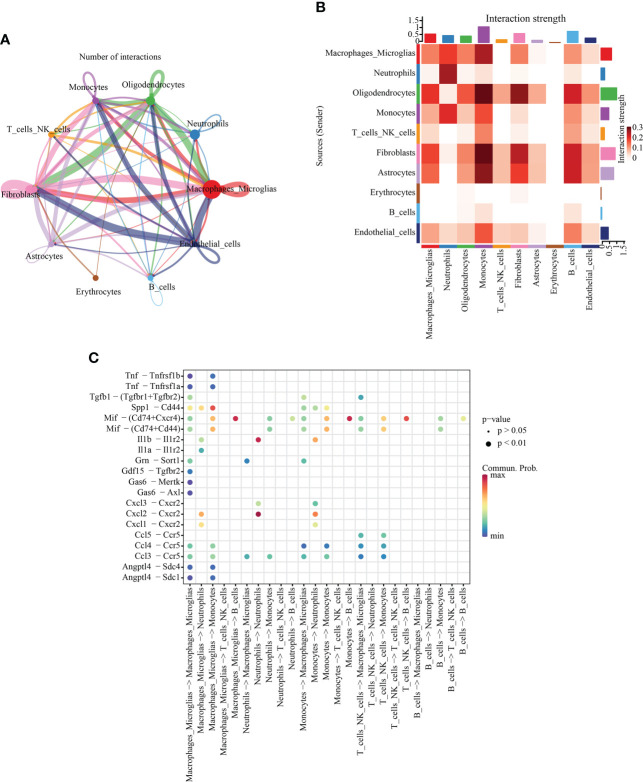
Intercellular communication analysis. **(A)** Quantitative network diagram in which the nodes represent different cell types, the arrows indicate the interaction signals from the ligand cell to the recipient cell, and the thickness of the line thickness indicates the number of significant ligand−receptor interaction pairs detected between different cell types. **(B)** Heatmap showing intercellular interaction intensity. The redder the color, the higher the proportion of interaction between ligand−receptor pairs. **(C)** The ligand−receptor pairs involved in intercellular communication between immune cells. The column represents the cell type of cellular communication (receptor cell ligand cell), the circle size indicates the significance level, and intensity of red color is directly proportional to the probability of communication between the interacting cells.

To further understand the communication relationship between cells, we used CellChat to analyze the intercellular receptor−ligand pairs and molecular interactions between the two cell types. CellChat analysis of ten cell states showed that macrophages/microglia were the dominant communication centers, and 58 pairs (ligand cells) and 96 pairs (receptor cells) were involved in cell interactions. The most obvious interaction with fibroblasts was 15 ligand−receptor pairs in ligand cells, and the most obvious interaction with fibroblasts (19 pairs) and endothelial cells (19 pairs) was observed in receptor cells (**Figure 5A**). The strength of interaction signal strength is shown by a heatmap (**Figure 5B**). We focused on the immune cell group, so we used a dot diagram to show the ligand−receptor pairs of intercellular communication among immune cells. Previous studies have shown that SCI leads to the accumulation of inflammatory cytokines in the injured areas, but in which cells it is not clear. The chemokine family can be subdivided into CXC and CC chemokine ligands (CXCL, CCL) (28). In the inflammatory response, the CCL family is mostly involved in monocyte recruitment, while the CXC family is mostly involved in neutrophil recruitment (29). Our results also confirm this point of view. The bubble chart shows that Cxcl3-Cxcr2 and Cxcl2-Cxcr2 may be autocrine from neutrophils, and Ccl5-Ccr5, Ccl4-Ccr5 and Ccl3-Ccr5 may be paracrine from macrophages/microglia (**Figure 5C**).

### IRAFs activation network of SCI

**Figure 6:**
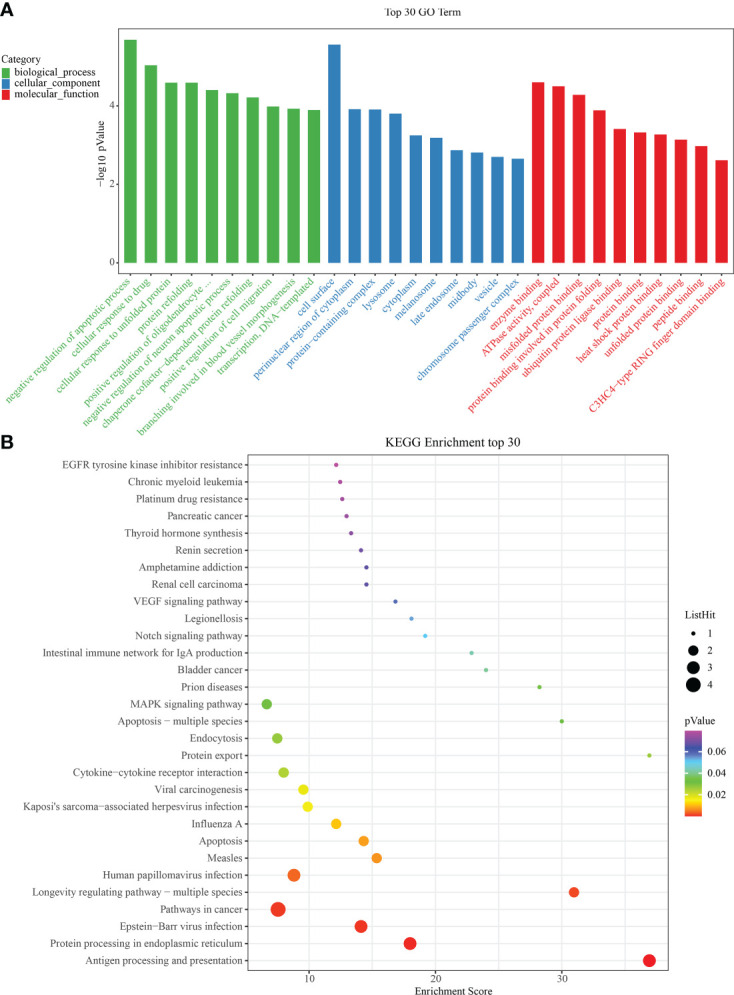
Gene ontology/Kyoto Encyclopedia of Genes and Genomes (GO/KEGG) enrichment results. **(A)** Bar graphs showing GO on BP (Biological Process), MF (Molecular Function) and CC (Cellular Component) levels. **(B)** Bubble diagram showing the enrichment results of KEGG analysis.

To identify significant enrichment of biological processes, we used clusterProfiler (30, 31) to conduct the GO/KEGG enrichment analysis of the IRAFs to identify the significantly enriched biological processes. The first ten terms of the three major functional categories were selected for visualization with a column chart. IRAFs were significantly enriched in cell response to drugs and positive regulation of cell migration and other biological processes. The main cellular components involved are the cell surface, lysosome and perinuclear region of the cytoplasm. The main molecular function was enzyme binding (**Figure 6A**). Similarly, the significance threshold of KEGG enrichment analysis was set to p < 0.05. It was arranged in ascending order according to the P value, and the first 30 pathways are shown in the bubble chart, which are enriched in the pathways related to MAPK, Notching and apoptosis (**Figure 6B**). Gene set variation analysis results also showed that injury activated the MAPK and Notching signaling pathway (**Supplementary Figure S6**).

### Study of the IRAFs expression pattern and immune cell distribution

**Figure 7:**
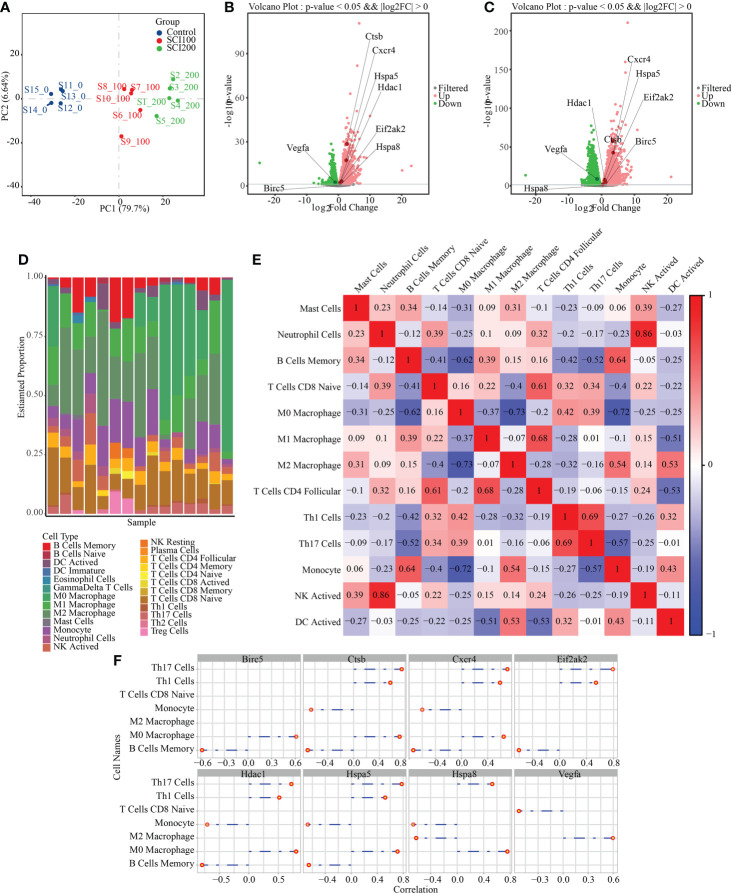
Difference analysis and immune cell prediction of RNAseq data. **(A)** principle component analysis map of the GSE115067 dataset. **(B)** Volcanic map of differences between groups, in which the differential expression of IRAFs is marked; SCI100-vs- Uninjured. **(C)** SCI200-vs-Uninjured. **(D)** Accumulation map of immune cell type score. **(E)** Correlation heat map of immune cells, where red represents positive correlation, blue represents negative correlation, and white represents no correlation. Darker red or blue color represents a stronger correlation. **(F)** Visualization of the correlation between IRAF expression and immune cells.

To study the expression of IRAFs at the tissue level in SCI, we analyzed the similarities and differences between samples and groups using GSE115067 and PCA of transcriptome sequencing data sets (**Figure 7A**). The differences between the two comparison groups were analyzed by calling the DESeq2 package: 100 kdyn percussion SCI disease group and uninjured group (SCI100-vs-Uninjured), 200 kdyn impact SCI disease group and uninjured group (SCI200-vs-Uninjured). We set the significant differential gene screening threshold FoldChange to 1with a P < 0.05 and found that 4350 and 6429 genes were differentially expressed between the SCI100-vs-Uninjured and SCI200-vs-Uninjured, respectively. Volcano plots were used to display IRAFs (**Figure 7B, C**).

We first replaced the homologous genes of rats and mice, used a mouse immune cell matrix, and calculated the type and distribution of immune cells in RNA-seq data using the CIBERSORT algorithm.

Immune cells types with zero abundance in more than half of the samples were excluded, and Pearson correlation heatmaps among 13 expressed immune cell types were constructed (**Figure 7E**). We found a correlation between immune cells, a positive correlation between neutrophil cells and NK cells (r = 0.86), and a negative correlation between M0 Macrophages and M2 Macrophages (r = 0.73), M0 Macrophages and Monocytes (r = 0.72). A column chart was used to show the predicted results of the cell proportion (**Figure 7D**). For IRAFs, we also calculated the Pearson correlation between the expression of each gene and the score of immune cells, and drew the correlation coefficient lollipop map. Among them, M0 Macrophages were positively correlated with Ctsb (R = 0.71, p = 0.0028), Hspa8 (R = 0.76, p = 0.0011), and Hspa5 (R = 0.71, p = 0.0028). M2 Macrophages were positively correlated with Vegfa (R = 0.60, p = 0.019) and negatively correlated with Hspa8 (R = -0.62, p = 0.014) (**Figure 7F**).

## Discussion

SCI leads to the activation multiple biological processes that vary based on the cause, location, and severity of injury. Chance of survival and neural function, recovery, and motor ability vary based on the nature of the SCI (32). Additionally, cell activity is expected to change with stimulation (33). Based on the findings of our previous studies (26), different degrees of SCI have a different biological processes, and a significant positively and linearly correlation with the percentage of histological damage area and negatively significantly linearly correlation with the behavioral score, NeuN cell count, and spinal motor and sensory evoked potentials. Therefore, it is important to systematically characterize the cell lineage within each degree of SCI at the single-cell level. Autophagy (“self-eating”) is a process by which parts of the cell are transported to the lysosomal chamber for degradation and recycling (34, 35). Autophagy participates in a variety of biological activities, and functions in maintaining cell homeostasis (36). Studies have shown that autophagy is essential for the homeostasis of the central and peripheral nervous systems (37, 38). However, the exact molecular mechanism of autophagy in SCI at the single-cell level remains unclear. In this study, we constructed a single-cell map for different degrees of SCI for the first time in rats, screened eight related autophagy-immune related molecules, described the complex changes of cellular components at the site of SCI and confirmed the nature of cell−cell interactions.

SCI destroys the vascular system of the local spinal cord, resulting in hematoma, vasoconstriction, hypoperfusion, and ischemia (39). This can result in an imbalance in cell ion homeostasis and lead to further cell injury. Subsequently, the infiltration of peripheral inflammatory cells and the release of reactive oxygen species further aggravates the damage (4, 40). SCI activates reactive astrocyte proliferation (41), which gather at the injured site to form a fibrotic scar boundary and prevents axonal regeneration (42, 43). Macrophages can promote tissue repair by regulating the transformation at different stages of wound healing. Neonatal microglia and treated adult microglia can significantly improve healing and axonal regeneration (44). We captured a total of 56,287 cells, of which 1,191 (2.116%) were annotated as astrocytes and 29,197 (51.872%) were annotated as macrophages/microglia. This is basically consistent with the proportion of microglia reported previous (22). Differences between the groups of these cells may lead to different pathophysiological states.

Axonal regeneration of the injured central nervous system after injury is affected by immune cells (43). Cellular activity can be characterized by molecular and their interactions within the cells, so is similar to the process of signal transmission (45). Autophagy participates in intercellular communication, mediates the secretion of nonclassical proteins (46), and regulates the function of immune cell function (47). Immune response is a dynamic process involving active cells (48). We focused on immune-related and autophagy-related genes, and finally obtained eight IRAFs: Hdac1, Cxcr4, Ctsb, Birc5, Hspa5, Hspa8, Vegfa, and Eif2ak2. Cathepsin B (Ctsb) can be released from damaged lysosomes (49) and directly participate in the implementation of autophagy (50). Dysfunctional Ctsb genes can induce cell death (51). Macrophages play an important role in many inflammatory diseases. Ctsb expression in macrophages is involved in joint destruction and bone injury (52). Ctsb released by infiltrating macrophages promotes fibroblast activation and subsequent collagen deposition (53). Similarly, a link has been found between CTSB and the microglia of Alzheimer’s disease and amyotrophic lateral sclerosis in mice (54). However, until now the role of CTSB expression in SCI has been unclear. Using single-cell sequencing, we observed a significant increase in the expression of Ctsb in macrophage/microglia induced by SCI, which may be related to the activation of autophagy. Therefore, inhibition of Ctsb expression may be a potential therapeutic strategy for the treatment of SCI.

Cell surface receptors activate the mitogen-activated protein kinase (MAPK) cascade, which consists of a three-tiered module of MAPKKK, MAPKK and MAPK (55). At present, the role of the complex MAPK signaling pathway in the regulation of autophagy after SCI has not been fully described, and describing the signal cascade of autophagy and its mechanism will therefore be highly beneficial to the treatment and prevention of SCI. In eukaryotic signal transduction, many MAPK pathways are intertwined with autophagy, including the MAPK/ERK, MAPK/JNK and MAPK/p38 pathways (56). MAPK/ERK activity plays an active role in autophagy (57), which is stimulated by direct interaction with autophagy-related proteins (58). Studies have shown that autophagy and fibrosis induced by transforming growth factor β1 are reduced after inhibition of the ERK and JNK signaling pathways (59). The p38 MAPK pathway is the most important member of the MAPK family in the regulation of inflammation. JNK and p38MAPK are involved in mediating the responses of various extracellular stress stimuli and proinflammatory cytokines (60), such as CCL3-CCR1 and 5, CCL4-CCR1 and 5 and CCL5-CCR1,3 and 5 in macrophages (61), and CXCL2-CXCR2 in neutrophil (62). Interestingly, our results also show that he elevated expression of CCL3,4-CCR5 in macrophages/microglia, CCL5-CCR5 in cells/NK cells, and CXCL2-CXCR2 in neutrophils. In summary, our results reveal the role of immune-related autophagy in SCI, and we identified chemokines corresponding to different cells local to SCI.

In this study, we described the expression of autophagy-related genes at the single-cell level at the site of SCI in rats and discussed the potential mechanisms. However, owing to the limitations of single-cell sequencing, such as the inability to accurately describe low-expression genes, large sample sizes are required for reliable analysis. Additionally, owing to the lack of corresponding clinical specimen research, it cannot be analyzed in combination with clinical information. We may conduct future research in this direction using multigroup science and space transcriptome technology.

In summary, our scRNA-seq dataset is the first full transcriptional analysis of SCI in rats, and it encompasses almost all the cells in the region of SCI. Through this dataset, we not only evaluated the heterogeneity of the cells that make up the injured site, but also screened the signal pathways in which IRAFs interact with each other at the injured site. Our analysis revealed new insights into the effect of immune cells on cellular heterogeneity, and the role of specific signaling pathways in autophagy in injured tissues. These results can help decipher the pathophysiological basis of SCI, which is difficult to treat.

## Data availability statement

The datasets presented in this study can be found in online repositories. The names of the repository/repositories and accession number(s) can be found in the article/**Supplementary Material**.

## Ethics statement

The animal study was reviewed and approved by All experimental procedures were approved by the Animal and Ethics Committee of the Experimental Animal Center of Air Force Medical University (No. IACUC-20201003).

## Author contributions

LEL, RY, and KY contributed to all steps of the study and wrote the manuscript. RZ, HQ, and QZ participated in rat modeling. PZ and HQ participated in Immunofluorescence and transmission electron microscopy experiments. HW participates in single-cell data analysis, and SL participated in statistical analysis. BL and QM contributed to the project design. All authors contributed to the article and approved the submitted version.

## Funding

This work was financially supported by the National Natural Science Foundation of China (No. 82174166), and the Innovation Capability Support Program of Shaanxi (No. 2021TD-45).

## Conflict of interest

The authors declare that the research was conducted in the absence of any commercial or financial relationships that could be construed as a potential conflict of interest.

## Publisher’s note

All claims expressed in this article are solely those of the authors and do not necessarily represent those of their affiliated organizations, or those of the publisher, the editors and the reviewers. Any product that may be evaluated in this article, or claim that may be made by its manufacturer, is not guaranteed or endorsed by the publisher.
