# Supplementary material for: Single-cell RNA sequencing reveals the role of immune-related autophagy in spinal cord injury in rats
Source: Front Immunol. 2022 Sep 21;13:987344. doi: 10.3389/fimmu.2022.987344 (PMC9535363; doi:10.3389/fimmu.2022.987344)
Supplement: Supplementary file 1 [file DataSheet_1.pdf]

Supplementary Materials and Methods

Animals and breeding

For sequencing, and histological validation of tip cells, female Wistar rats [SPF Biotechnology Co., Ltd., Beijing, China. certificate no. SCXK (Jing) 2019-0010] were reared in the Orthopaedic Laboratory of the second affiliated Hospital of Air Force military Medical University [certificate no. SCXK (Shaanxi) 2020-007]. The feeding conditions are as previously described(Yan et al., 2022a). All experimental procedures were approved by the Animal and Ethics Committee of the Experimental Animal Center of Air Force Medical University (No. IACUC-20201003).

SCI surgical procedures

All animals were randomly divided into the following groups, the randomization method as previously described(Yan et al., 2022a), the sham group, the mild group, the moderate group, and the severe group. The rats were anesthetized by intraperitoneal injection of 3% sodium pentobarbital (40 mg/kg). Surgical procedures were described previously(Bellver-Landete et al., 2019; Liu et al., 2021). Briefly, according to the special anatomical structure of T9-T11 spinous process, T9 was determined, T9 spinal cord was exposed, and bilateral pedicles were fixed. Above T9, the weight (10g) was contused from 12.5mm 25mm and 50mm height respectively, resulting in mild, moderate and severe injury. Rat lower limb trembling and tail droop indicate that the model is successful. In the sham operation group, all the animals underwent laminectomy alone. After rinsing with normal saline, suture the skin layer by layer. Throughout the whole surgery, the body temperature of the rats was maintained at approximately 37 °C using a heating pad. All procedures were performed by one experimenter, while the impactor was operated on by another experimenter. After the surgery, the bladders of the rats were massaged twice daily until urination function was restored(Yao et al., 2018). Buprenorphine (0.05 mg/kg) was injected subcutaneously daily for 3 days, and cefazolin (50 mg/kg) was administered subcutaneously once daily for 7 days. From the beginning to the end of the experiment, the general condition of the rats was assessed, and wounds, infections, and other alterations were monitored daily. Animals were excluded if they died prematurely.

Tissue preparation for scRNA-seq

The rats were deeply anesthetized with pentobarbital (100 mg/kg) at 7 dpi, and 10-mm spinal cord tissue (from 5 mm rostral to 5 mm caudal from lesion core) was harvested on ice. Single cell dissociation and sequencing were completed with the assistance of LC-Bio Technology (Hangzhou, China). Briefly, first, single cells were separated by 10x genomics platform microfluidic technology, and then the gel beads with barcode and primers and a single cell were wrapped in oil droplets; the gel beads dissolved in the oil droplets to release reverse transcription oligo, and the cells cleaved to release mRNA. After the liquid oil layer of cDNA; with barcode was obtained by SMART method, the cDNA library was constructed, and finally the Illumina sequencing platform was detected.

We also downloaded the RNAseq dataset GSE115067(Squair et al., 2018) as an auxiliary analysis, the data sets are all samples derived from Rattus norvegicus, and the detection platform is NextSeq 550 (Rattus norvegicus). The data set we selected were sequenced samples of rats with different severity of SCI, including five biological repeats of 0,100,200 kdyn shock. A total of 15 samples were included in this study.

Quality control, normalization, and data integration

We used Seurat (version 4.0) R package(Hao et al., 2021) to import the Cellranger (version 6.0.1)(Zheng et al., 2017) result matrix of 8 samples of single-cell data set, and created the Seurat object for this analysis.

The proportion of mitochondrial genes to all genetic material may indicate whether the cell is in a steady state. We generally think that when the proportion of mitochondrial genes in a cell is higher than all genes, it may be in a state of stress. As a result, we filtered cells with mitochondrial gene content more than 10%. We also filter the twins according to the doublet ratio of the 10x genomics platform according to the standard of 0.077 * sample cell number, and filter outliers by 2 * (mean + /-standard deviation), that is, low-quality cells that specify the number of genes and the number of UMI. After the above steps, we obtained 56287 cells.

Standardize the data using the LogNormalize method. After controlling the relationship between average expression and dispersion, highly variable genes were identified in a single cell. Then, we use principal component analysis (Principal Component Analysis, PCA), using variable genes as input, and identify significant principal components according to the ElbowPlot function. The first 15 principal components were selected as the statistically significant input of t-Distributed Stochastic Neighbor Embedding (t-SNE).

Cell clustering, cell marker, and cell type identification

The FindClusters function was used to cluster the cells, and the resolution parameter was set to 0.4, which is divided into 22 clusters. We further looked for the differences between cell clusters, marker. In order to verify our cell type annotation, we first replaced the homologous genes of rats and mice and annotated the standard cell types by using the MouseRNAseqData data set in SingleR (version 1.8.1) R package(Aran et al., 2019) and related literature(Milich et al., 2021).

Single cell subgroup analysis and quasi-sequential analysis

Subset function was used to extract immune cell groups (macrophages/microglia, monocytes, neutrophils, T_NK cells, B cells), macrophages/microglia, T_NK cells for subpopulation analysis. The differences of macrophage/microglia subsets between mild injury group and control group, moderate injury group and control group, severe injury group and control group were analyzed, and the significant difference gene screening threshold FoldChange was set as 1pvalue less than 0.05.

Monocle (version 2.22.0) R packet(Qiu et al., 2017) was used to infer the cell differentiation of the above three subsets. The integrated gene expression matrix from each cell subgroup is first derived from the Seurat object to Monocle to construct the cell dataset. Use the variable gene defined by the dispersionTable function, and then use the setOrderingFilter function to sort the cells.

The DDRTree method is used to reduce the dimension and the orderCells function is used to estimate the arrangement of cells along the trajectory. Based on the clustering characteristics and marker gene analysis, the trajectory map of the differentiation time of immune cells in single cell data set was obtained. The analysis of each track adopts a standard scheme with default parameters.

GO and KEGG enrichment analysis and Gene set variation analysis

Gene Ontology (GO)enrichment analysis is a common method for large-scale functional enrichment of genes in different dimensions and different levels. Fisher accurate test is used to calculate the enrichment significance of each Term in biological processes (BP), molecular function (MF) and cellular components (CC), and count the number of differential genes included in each GO entry. The hypergeometric distribution algorithm(Mi et al., 2017) was used to calculate the significance of differential gene enrichment in each GO entry. The calculation results will return a P value of rich significance, and the smaller this value, the more significant it is statistically.

Kyoto Encyclopedia of Genes and Genomes (KEGG) enrichment analysis uses hypergeometric distribution

to calculate the correlation between each pathway in KEGG pathway and this differential gene. The smaller the P-value, the correlation degree between the corresponding pathway and this differential gene.

Gene set variation analysis (GSVA) Analysis of immune Cell Group in single Cell data set based on R language GSVA package(Hänzelmann et al., 2013). GSVA analysis carried out rank statistics calculation similar to K-S test for the gene set corresponding to each pathway. Then the pathways with significant differences were obtained by limma package(Ritchie et al., 2015), so as to evaluate the enrichment degree of different pathways among different groups. Statistical significance was defined as $P < 0.05$.

Analysis of intercellular communication

CellChat (version 1.1.3) R packeage(Jin et al., 2021) is often used to analyze intercellular communication networks from single-cell rna sequencing data. CellChat was used to quantitatively infer and analyze the intercellular communication network from single-cell rna sequencing data, circle map was used to show the interaction of cell populations, and the bubble diagram was used to count all the important ligand pairs in intercellular signal transmission.

Analysis of cell score and Regulon regulator based on immune-related autophagy factors

We focus on autophagy genes related to immunity, so we download immune genes and autophagy genes from ImmPort database (Bhattacharya et al., 2014) (https://www.immport.org/) and HADb human autophagy database (http://www.autophagy.lu/index.html) for follow-up analysis. Based on our research species. Firstly, rat homologous genes were screened from the list of human immune genes and human autophagy genes by homologene (version 1.1.3) R package (https://oganm.github.io/homologene/). The differential immune genes were obtained in the difference analysis of immune cell subsets, and then through the intersection of these differential immune genes and autophagy-related homologous genes, the immune-related autophagic factors (IRAFs) of rats were finally obtained.

Based on the above IRAFs dataset, we use the AddModuleScore function to calculate the score of a single cell in this gene set. Then all the cells were divided into high and low group cell groups according to the median score, and finally the diseased sample cell groups in the high score cell group were selected for follow-up SCENIC analysis.

Multicellular organisms contain a variety of cell types, each with its own morphology and function. Cell types are generally maintained by the coordination and interaction between transcription factors and their corresponding target genes. We can identify the co-expressed modules (regulon) between transcription factors (TFs) and potential target genes and the regulon activity scores of each cell (regulon activity score, RAS) through SCENIC (version 1.1.2)(Aibar et al., 2017) software.

Analysis of differences between groups based on RNA-seq data

We download the Counts matrix of the RNAseq dataset, convert Ensembl ID to GeneSymbol, and organize the matrix file. DESeq2 (version 1.34.0) R packet(Love et al., 2014) was used to analyze the difference between the uninjured group and the injured group. The significant difference gene screening threshold FoldChange was set as 1m p value less than 0.05p value. PCA principal component analysis is used to visualize the overall distribution of samples.

Estimation of the fraction of immune cell types

CIBERSORT (https://cibersortx.stanford.edu/)(Newman et al., 2015) analysis tool is a bioinformatics algorithm for accurate calculation of immune cell infiltration. The hypothetical immune cell abundance was estimated using a reference set of 25 immune cell subtypes (including Mast Cells, Neutrophil Cells, Eosinophil Cells, B Cells Memory, B Cells Naive, Plasma Cells, T Cells CD8 Actived, T Cells CD8 Naive,

T Cells CD8 Memory,M0 Macrophage,M1 Macrophage,M2 Macrophage, Treg Cells, T Cells CD4 Memory, T Cells CD4 Naive, T Cells CD4 Follicular,Th1 Cells,Th17 Cells,Th2 Cells, Monocyte, GammaDelta T Cells, NK Resting, NK Actived, DC Actived, DC Immature) and 1000 permutations. Use the R software to set the number of combinations to 1000. A bar chart was drawn to show the composition of infiltrating immune cells in each sample. Corrplot ((https://github.com/taiyun/corrplot) version 0.92) R packet was used to visualize the pearson correlation analysis of 25 kinds of infiltrating immune cells.

qPCR

Real-time quantitative PCR (RT-qPCR) was performed using 2qPCR SmArt Mix (DIYIBio, Shanghai, China). Three primer sets were tested for each gene and showed identical results. Primer sequences are listed in Table 1. Quantitative PCR was performed in a Rotor-Gene Q with SYBR reagent in a final volume of 20 μL per well. Samples were amplified independently at least three times. Relative gene expression was converted using the $1.8^{-\Delta\Delta Ct}$ method against GAPDH. GAPDH primer: forward,5'-ACAGCAACAGGGTGGTGGAC-3' and reverse, 5'-TTTGAGGGTGCAGCGAACTT-3'. Hdac1 primer: forward, 5'-TTCTGTCAGTTGTCCACGGG-3' and reverse, 5'-AACAGAAGCCGGATGCTTCA-3'. Cxcr4 primer: forward, 5'-GCCATGGCTGACTGGTACTT-3' and reverse, 5'-CACCCACATAGACGGCCTTT-3'. Ctsb primer: forward, 5'-CAGGCTGGACGCAACTTCTA-3' and reverse, 5'-TCTGATCTGTGCGATGGTCG-3'. Birc5 primer: forward, 5'-CTTCATCCACTGCCCTACCG-3' and reverse, 5'-GGGGAGTGCTTCCTATGCTC-3'. Hspa5 primer: forward, 5'-TCGACTTGGGGACCACCTAT-3' and reverse, 5'-AGTGAAGGCCACATACGACG-3'. Hspa8 primer: forward, 5'-CTTGGCACCACCTACTCCTG-3' and reverse, 5'-GAAAGCAACATAGCTCGGCG-3'. Vegfa primer: forward, 5'-CAAACCTCACCAAAGCCAGC-3' and reverse, 5'- TTCTCCGCTCTGAACAAGGC-3'. Eif2ak2 primer: forward, 5'-AGCAGAACTCAATCACGCCA-3' and reverse, 5'- GGATAAAGAGGCACCGGGTC-3'.

Transmission Electron Microscopy (TEM)

After the fresh tissue determines the material selection part, cut the required observation sample into small pieces of about $1mm^3$. Fix in 2.5% glutaraldehyde at 4℃ for 2-4h. The tissue block was rinsed three times with 0.1M phosphoric acid rinse solution for 15min each time, and then the sample was placed in a refrigerator with 1% osmic acid at 4℃ and fixed for 2h. The sample is added into 30%-50%-70%-80%-95%-100%-100% alcohol in turn for gradient dehydration, 15min each time, 100% propylene oxide for 3 times, 5 min each time. Propylene oxide + embedding solution (2: 1) at room temperature for 2h, Propylene oxide + embedding solution (1: 2) at room temperature for 3h, and pure embedding solution at room temperature overnight. Replace the pure embedding solution at room temperature for 3-4h. Then pick out the sample with toothpick and embed it on the embedding plate, Placing the embedding plate in an oven at 60℃ for 48h, and taking out the embedding block for later use after the resin is completely polymerized. After rough trimming, the resin block is semi-thin sliced in an ultra-thin slicer, and after trimming according to the positioning condition, the required position is ultra-thin sliced with a slice thickness of 70nm, and the copper net is used for fishing. Dyeing with 3% uranium acetate saturated alcohol solution for 8 min; 70% alcohol for 3 times and ultrapure water for 3 times; Staining with 2.7%

lead citrate solution for 8 min; Clean with ultrapure water for 3 times, and slightly dry the filter paper. Observation and photographing under transmission electron microscope (HITACHI- HT7700, Japan).

Immunostaining

Immunofluorescence staining have been detailed in previous publications(Yan et al., 2022b). Briefly, The sections were incubated, rinsed, antigen-retrieval, permeabilized, blocked, and then incubated with primary antibody overnight at 4 ℃ in a wet box. The following primary antibodies were used: rabbit anti-CD68 (1:200, Abcam, Cambridge, UK), mouse anti-Lc3b (1:100, Abcam), After three rinses in PBS for 10 minutes each, the sections were incubated with Coralite488-conjugated goat anti-rabbit IgG (1:200, Proteintech, Wuhan, China) or Cy3-conjugated goat anti-mouse IgG (1:100, Proteintech) for 1 hour at room temperature. The sections were washed in PBS 3 times and then coverslipped with a drop of anti-fade DAPI-Fluoromount G (SouthernBiotech, Birmingham, AL, USA) for nuclear counterstaining. The sections were visualized with an inverted fluorescence microscope BX51, and the images were analyzed with ImageJ software.

Supplementary figures

figure S1, Data quality control. A, Quality control (QC). B, nCount (UMI). C, nCount (Gene). D, Percent of cell in Clusters

figure S2, In the immune cell group, the immune-related intersection genes were obtained by the analysis of differences between groups, which were shown in the violin picture.

figure S3, Quasi-chronological analysis. A, Based on the pseudo-sequence diagram of each cell cluster during the differentiation process. B, Quasi-time trajectory map, the color from dark to light, indicating the time of differentiation from early too late.

figure S4, Heat map of Transcription Factors regulation intensity. A, Regulon regulons analyze the RAS-specific heatmap of regulons in each cell cluster, with rows indicating different regulons and columns indicating different cell clusters. The color changes from blue to red indicating that the RAS activity score ranges from low to high, and the RAS score indicates that the stronger the activity of regulon in this cell population.

figure S5, Lc3b was upregulated in Macrophages/microglia after SCI. A, Double immunostaining analysis of Lc3b (green) and Cd68 (red) in horizontal sections of uninjured spinal cords and injured spinal cords at mild, moderate and severe degree. B, Quantitative analysis of the relative fluorescent intensity of Lc3b level (normalized to sham group) at different degrees after SCI as shown in (A) ($n = 3$ per group). Scale bars, 200 μm. Data were mean ± SD. One-way ANOVA with Bonferroni's post-tests, *$P < 0.05$, ****$P < 0.0001$.

figure S6, Gene set variation analysis.

Supplementary table
Table S1, Summary of cell marker gene

Supplementary References

Aibar, S., González-Blas, C.B., Moerman, T., Huynh-Thu, V.A., Imrichova, H., Hulselmans, G., et al. (2017). SCENIC: single-cell regulatory network inference and clustering. *Nat Methods* 14(11)**,** 1083-1086. doi: 10.1038/nmeth.4463.

Aran, D., Looney, A.P., Liu, L., Wu, E., Fong, V., Hsu, A., et al. (2019). Reference-based analysis of lung single-cell sequencing reveals a transitional profibrotic macrophage. *Nat Immunol* 20(2)**,** 163-172. doi: 10.1038/s41590-018-0276-y.

Bellver-Landete, V., Bretheau, F., Mailhot, B., Vallières, N., Lessard, M., Janelle, M.E., et al. (2019). Microglia are an essential component of the neuroprotective scar that forms after spinal cord injury. *Nat Commun* 10(1)**,** 518. doi: 10.1038/s41467-019-08446-0.

Bhattacharya, S., Andorf, S., Gomes, L., Dunn, P., Schaefer, H., Pontius, J., et al. (2014). ImmPort: disseminating data to the public for the future of immunology. *Immunol Res* 58(2-3)**,** 234-239. doi: 10.1007/s12026-014-8516-1.

Hänzelmann, S., Castelo, R., and Guinney, J. (2013). GSVA: gene set variation analysis for microarray and RNA-seq data. *BMC Bioinformatics* 14**,** 7. doi: 10.1186/1471-2105-14-7.

Hao, Y., Hao, S., Andersen-Nissen, E., Mauck, W.M., 3rd, Zheng, S., Butler, A., et al. (2021). Integrated analysis of multimodal single-cell data. *Cell* 184(13)**,** 3573-3587.e3529. doi: 10.1016/j.cell.2021.04.048.

Jin, S., Guerrero-Juarez, C.F., Zhang, L., Chang, I., Ramos, R., Kuan, C.H., et al. (2021). Inference and analysis of cell-cell communication using CellChat. *Nat Commun* 12(1)**,** 1088. doi: 10.1038/s41467-021-21246-9.

Liu, H., Zhang, J., Xu, X., Lu, S., Yang, D., Xie, C., et al. (2021). SARM1 promotes neuroinflammation and inhibits neural regeneration after spinal cord injury through NF-κB signaling. *Theranostics* 11(9)**,** 4187-4206. doi: 10.7150/thno.49054.

Love, M.I., Huber, W., and Anders, S. (2014). Moderated estimation of fold change and dispersion for RNA-seq data with DESeq2. *Genome Biol* 15(12)**,** 550. doi: 10.1186/s13059-014-0550-8.

Mi, H., Huang, X., Muruganujan, A., Tang, H., Mills, C., Kang, D., et al. (2017). PANTHER version 11: expanded annotation data from Gene Ontology and Reactome pathways, and data analysis tool enhancements. *Nucleic Acids Res* 45(D1)**,** D183-d189. doi: 10.1093/nar/gkw1138.

Milich, L.M., Choi, J.S., Ryan, C., Cerqueira, S.R., Benavides, S., Yahn, S.L., et al. (2021). Single-cell analysis of the cellular heterogeneity and interactions in the injured mouse spinal cord. *J Exp Med* 218(8). doi: 10.1084/jem.20210040.

Newman, A.M., Liu, C.L., Green, M.R., Gentles, A.J., Feng, W., Xu, Y., et al. (2015). Robust enumeration of cell subsets from tissue expression profiles. *Nat Methods* 12(5)**,** 453-457. doi: 10.1038/nmeth.3337.

Qiu, X., Mao, Q., Tang, Y., Wang, L., Chawla, R., Pliner, H.A., et al. (2017). Reversed graph embedding resolves complex single-cell trajectories. *Nat Methods* 14(10)**,** 979-982. doi: 10.1038/nmeth.4402.

Ritchie, M.E., Phipson, B., Wu, D., Hu, Y., Law, C.W., Shi, W., et al. (2015). limma powers differential expression analyses for RNA-sequencing and microarray studies. *Nucleic Acids Res* 43(7)**,** e47. doi: 10.1093/nar/gkv007.

Squair, J.W., Tigchelaar, S., Moon, K.M., Liu, J., Tetzlaff, W., Kwon, B.K., et al. (2018). Integrated systems analysis reveals conserved gene networks underlying response to spinal cord injury. *Elife* 7. doi: 10.7554/eLife.39188.

Yan, R., Li, E., Yan, K., Zhang, Q., Wen, Y., Zhang, R., et al. (2022a). A modified impactor for establishing a graded contusion spinal cord injury model in rats. *Annals of Translational Medicine*.

Yan, R., Li, E., Yan, K., Zhang, Q., Wen, Y., Zhang, R., et al. (2022b). A modified impactor for establishing a graded contusion spinal cord injury model in rats. *Ann Transl Med* 10(8)**,** 436. doi: 10.21037/atm-21-5851.

Yao, Y., Xu, J., Yu, T., Chen, Z., Xiao, Z., Wang, J., et al. (2018). Flufenamic acid inhibits secondary hemorrhage and BSCB disruption after spinal cord injury. *Theranostics* 8(15)**,** 4181-4198. doi: 10.7150/thno.25707.

Zheng, G.X., Terry, J.M., Belgrader, P., Ryvkin, P., Bent, Z.W., Wilson, R., et al. (2017). Massively parallel digital transcriptional profiling of single cells. *Nat Commun* 8**,** 14049. doi: 10.1038/ncomms14049.
