# Supplementary figures and images for: Single-cell RNA sequencing reveals the role of immune-related autophagy in spinal cord injury in rats
Source: Front Immunol. 2022 Sep 21;13:987344. doi: 10.3389/fimmu.2022.987344 (PMC9535363; doi:10.3389/fimmu.2022.987344)

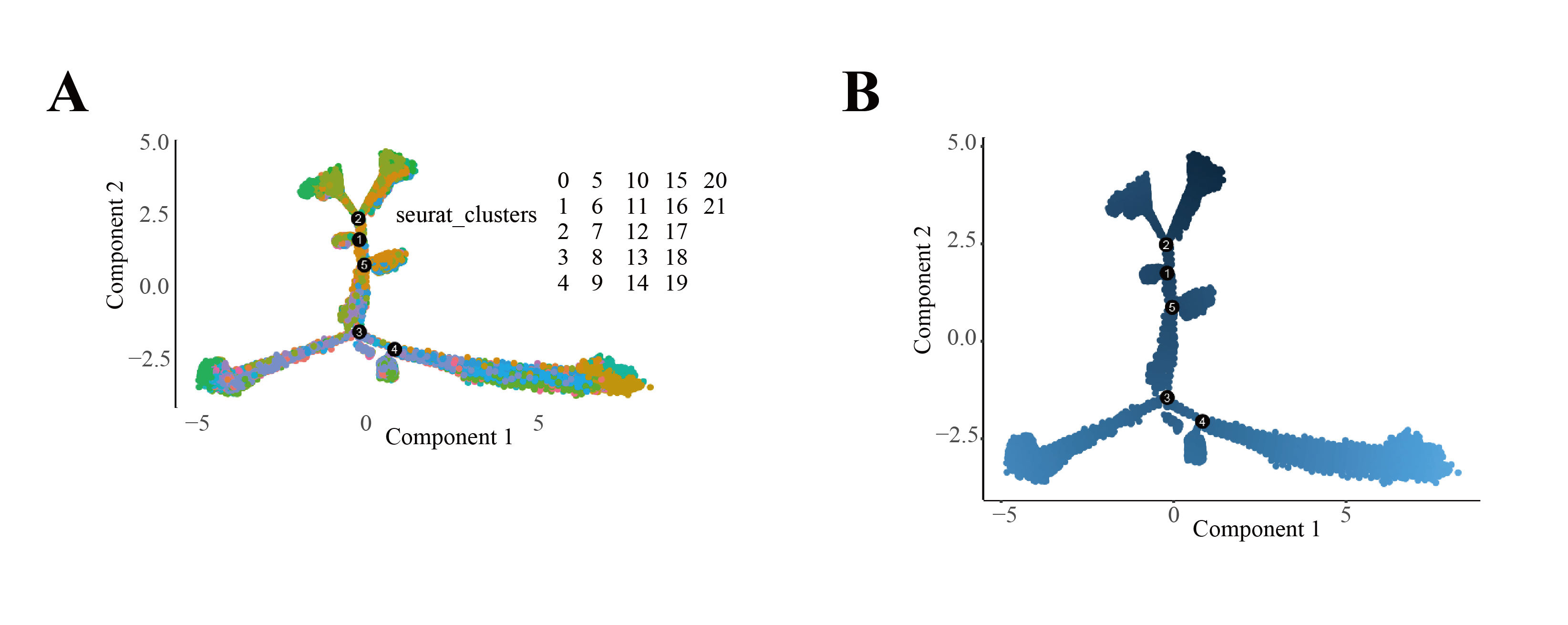

Supplement: Supplementary file 3 [file Image_1.tif]

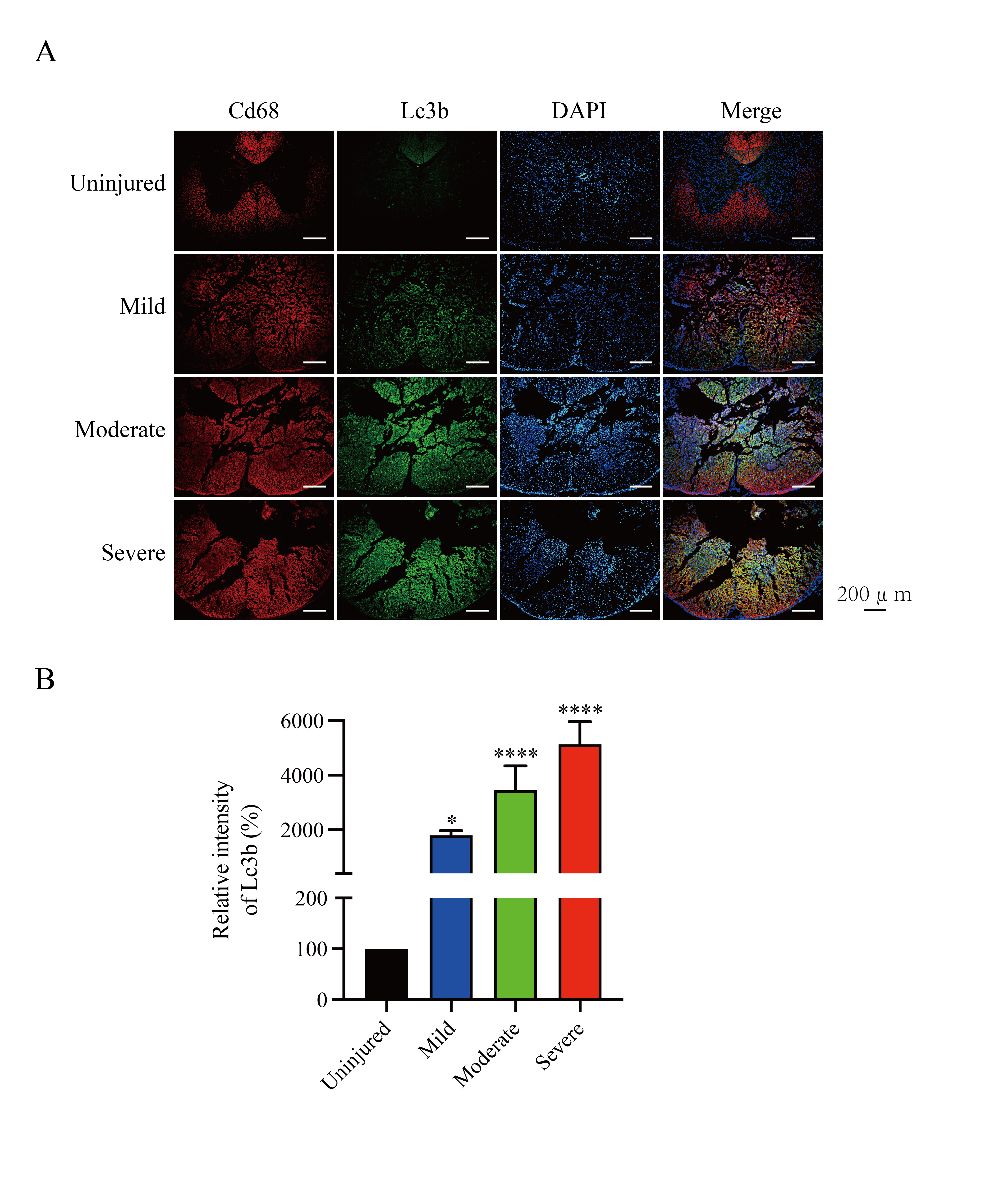

Supplement: Supplementary file 4 [file Image_2.tif]

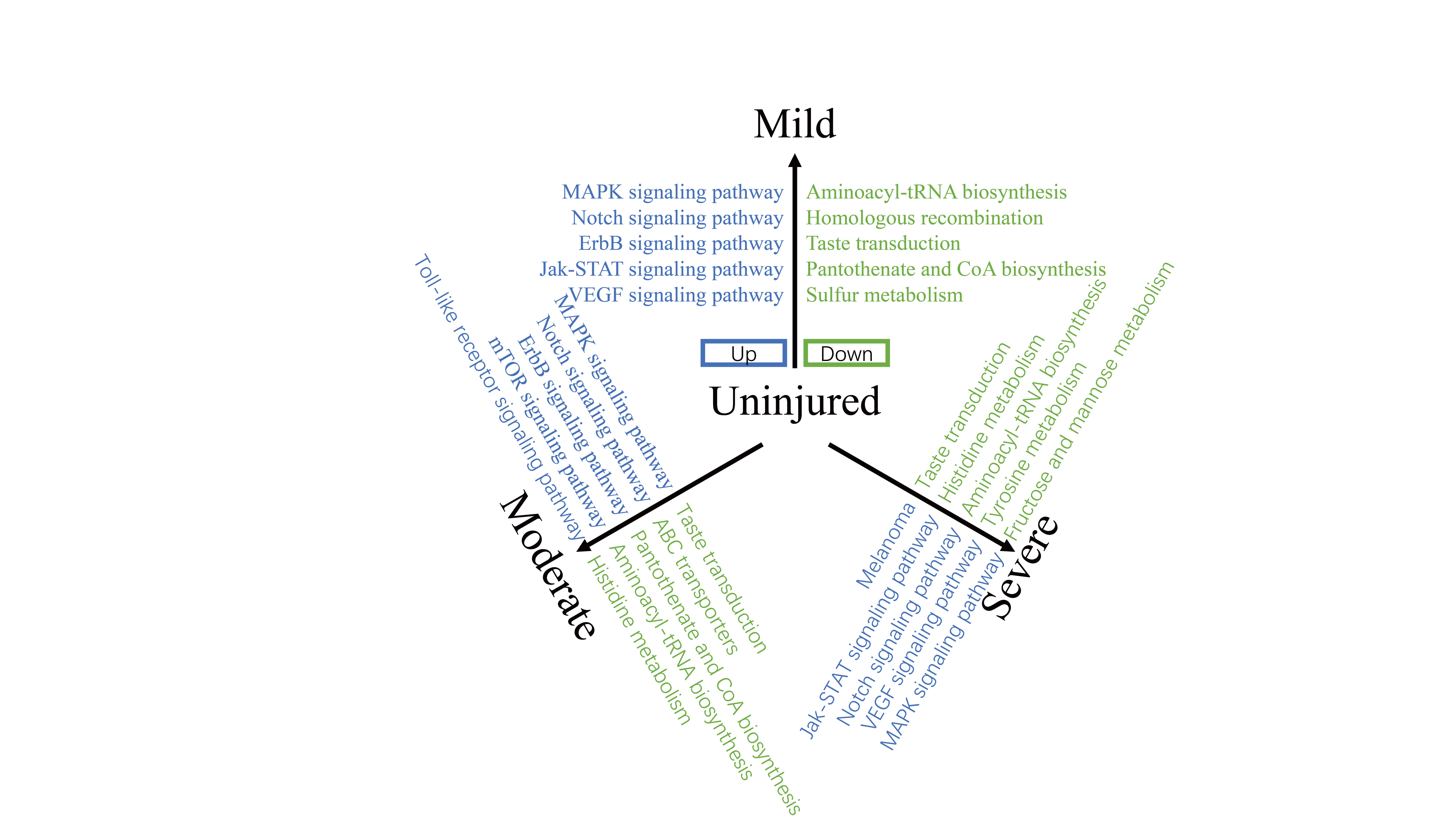

Supplement: Supplementary file 5 [file Image_3.tif]

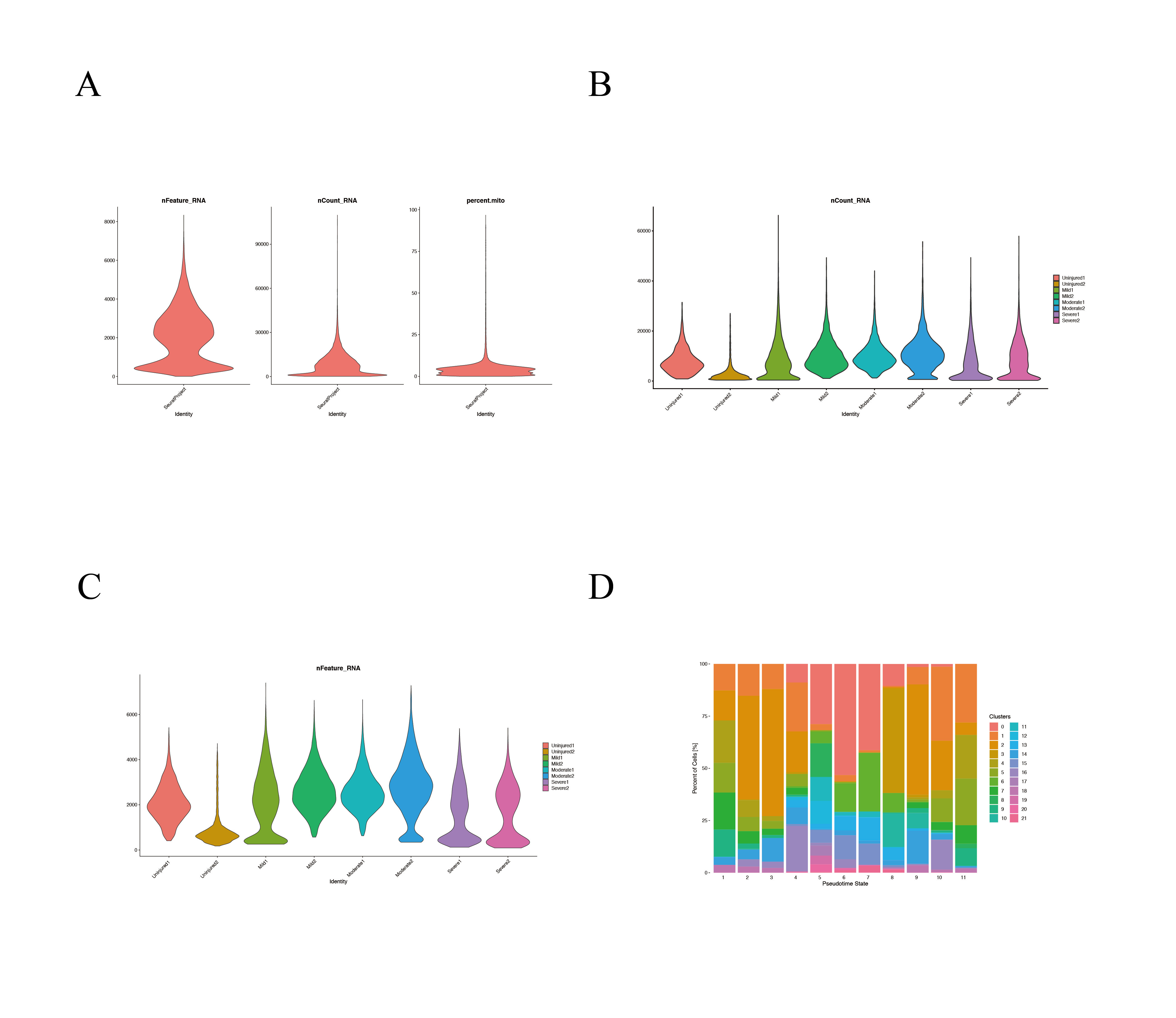

Supplement: Supplementary file 6 [file Image_4.tif]

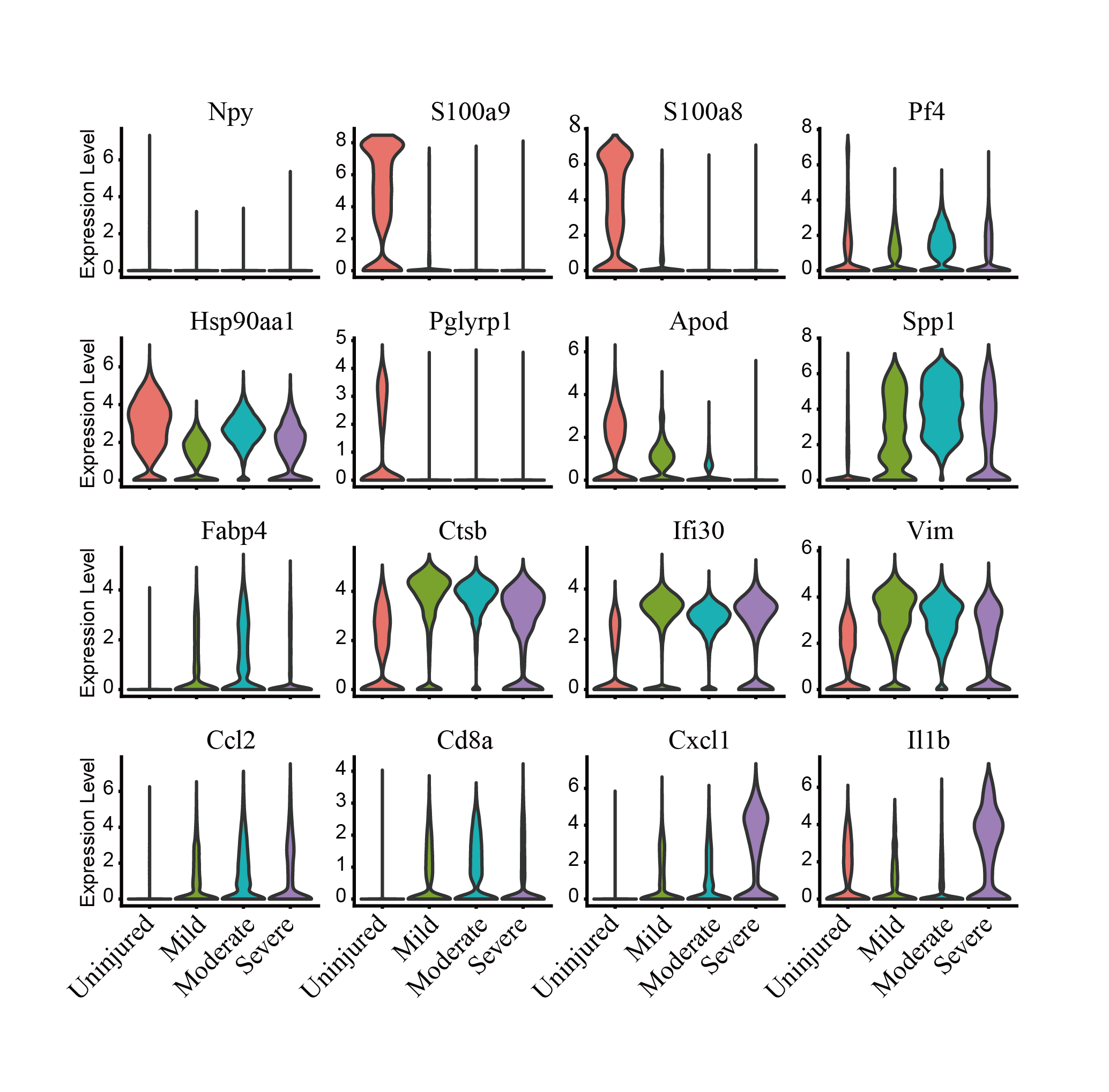

Supplement: Supplementary file 7 [file Image_5.tif]

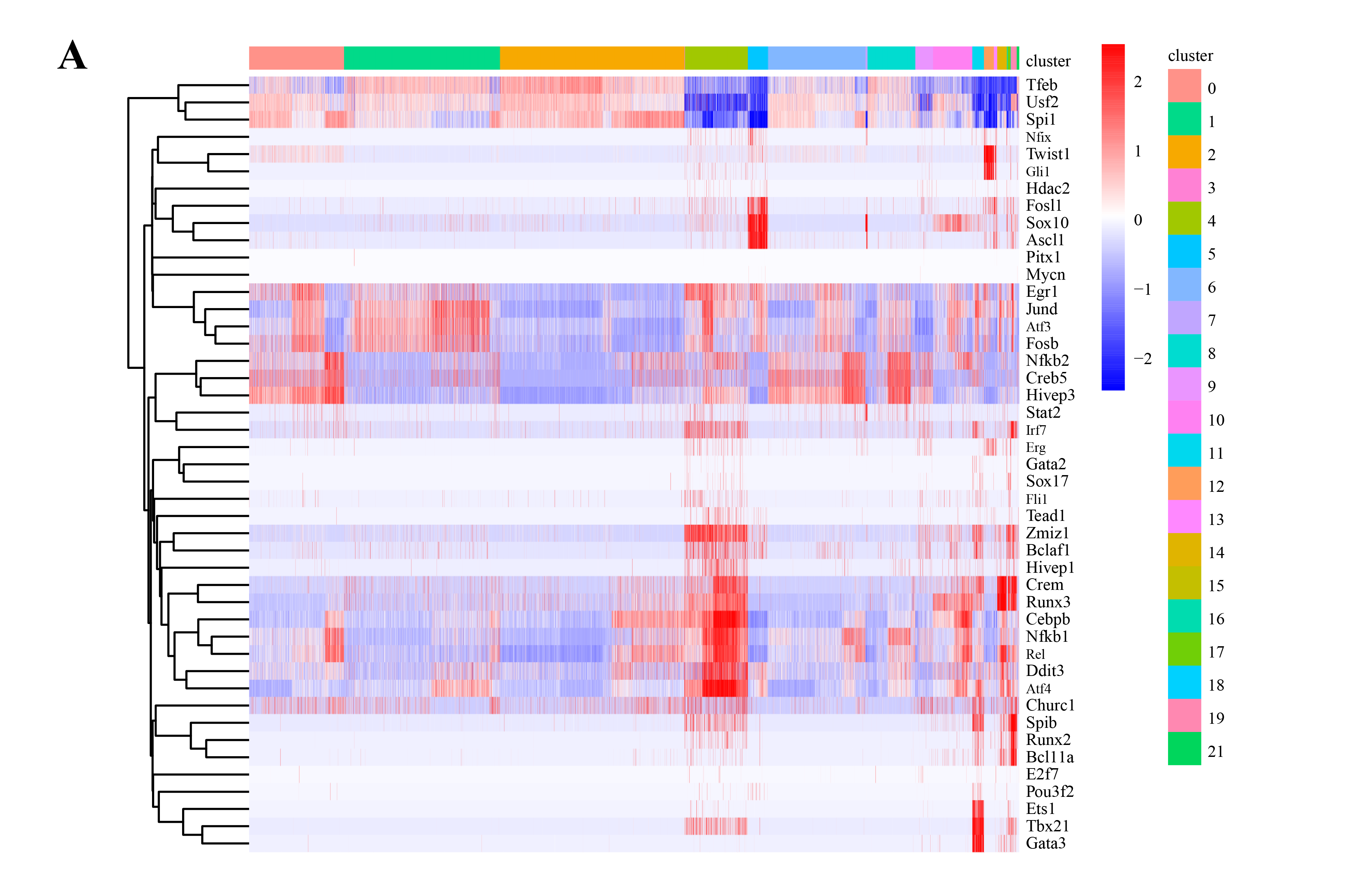

Supplement: Supplementary file 8 [file Image_6.tif]
